# A low-cost hierarchical nanostructured beta-titanium alloy with high strength

**DOI:** 10.1038/ncomms11176

**Published:** 2016-04-01

**Authors:** Arun Devaraj, Vineet V. Joshi, Ankit Srivastava, Sandeep Manandhar, Vladimir Moxson, Volodymyr A. Duz, Curt Lavender

**Affiliations:** 1 Physical and Computational Sciences Directorate, Pacific Northwest National Laboratory, 902 Battelle Boulevard, Richland, Washington 99354, USA; 2 Energy and Environment Directorate, Pacific Northwest National Laboratory, 902 Battelle Boulevard, Richland, Washington 99354, USA; 3 Department of Materials Science and Engineering, Texas A&M University, 3003 College Station, Texas 77843, USA; 4 Environmental Molecular Sciences Laboratory, Pacific Northwest National Laboratory, 902 Battelle Boulevard, Richland, Washington 99354, USA; 5 Advance Materials Products Inc. (ADMA), 1890 Georgetown Road, Hudson, Ohio 44236, USA

## Abstract

Lightweighting of automobiles by use of novel low-cost, high strength-to-weight ratio structural materials can reduce the consumption of fossil fuels and in turn CO_2_ emission. Working towards this goal we achieved high strength in a low cost *β*-titanium alloy, Ti–1Al–8V–5Fe (Ti185), by hierarchical nanostructure consisting of homogenous distribution of micron-scale and nanoscale *α*-phase precipitates within the *β*-phase matrix. The sequence of phase transformation leading to this hierarchical nanostructure is explored using electron microscopy and atom probe tomography. Our results suggest that the high number density of nanoscale *α*-phase precipitates in the *β*-phase matrix is due to *ω* assisted nucleation of *α* resulting in high tensile strength, greater than any current commercial titanium alloy. Thus hierarchical nanostructured Ti185 serves as an excellent candidate for replacing costlier titanium alloys and other structural alloys for cost-effective lightweighting applications.

## Abstract (web summary)

Lightweight materials with high strength are desirable for applications where they could reduce energy consumption. Here, the authors develop a low cost beta-titanium alloy that uses a hierarchical nanostructure of precipitates with different sizes to achieve high strength.

## Graphical abstract

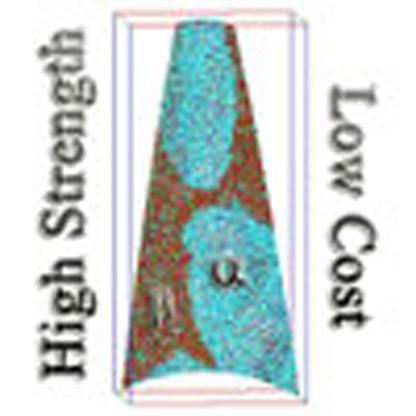

With rapid global climate change and alarming levels of atmospheric CO_2_ concentration, there is growing interest in and attention to reducing anthropogenic CO_2_ emission^1^,^2^. Emissions from fossil fuel combustion in transportation vehicles accounts for a significant portion of the global CO_2_ emissions^3^. This has recently led to development of number of government policies and mandates for global automobile manufacturers to meet increasingly stringent CO_2_ emission standards and higher fuel efficiency targets for future years^4^. Lightweighting of transportation vehicles is the key to reducing fossil fuel consumptions and anthropogenic CO_2_ emissions^1^,^2^,^3^. The primary approach to lightweighting is through increased use of structural materials with high specific strength (strength to weight ratio). In recent years, titanium alloys, more specifically *β*-titanium alloys, have been widely explored as candidate materials owing to their attractive specific strength, toughness and corrosion resistance^5^,^6^,^7^, for example, the Boeing 787 Dreamliner, which is one of the most fuel efficient airplanes in its class, is utilizing about 15% titanium alloys, the largest percentage of titanium used in a passenger airplane till date^8^. However, wide scale adoption of *β*-titanium alloys in transportation applications has been limited due to its high cost.

The cost of *β*-titanium alloys can be lowered by replacing the expensive *β* stabilizers such as Mo, Cr and V (in full or in part) by low cost Fe (ref. 9) such as the case in Ti–1Al–8V–5Fe (Ti185) alloy introduced in 1960s (ref. 10). Ti185 alloy demonstrated high tensile and shear strength proving to be an excellent candidate for fastener applications. Despite the advantages, the production of Ti185 alloy in bulk by conventional ingot processing remained a challenge since addition of Fe in excess of 2.5 wt% in titanium alloys lead to segregation of Fe. The segregation of Fe results in the formation of inhomogeneous *β* structures also known as *β* flecks, which is detrimental to the mechanical performance of the alloy^11^.

Recently the authors have demonstrated a novel route to develop engineering components of *β*-titanium alloys using a low-cost TiH_2_ powder feedstock^12^,^13^,^14^,^15^. The mechanical properties of the Ti185 alloy developed through this low-cost process are found to be at par with ones developed via the conventional route. The strength of the low-cost powder processed Ti185 alloy is further improved to achieve strength level higher than all current commercial titanium alloys by achieving a hierarchical nanostructure. The sequence of phase transformation leading to the formation of this hierarchical nanostructure and its role in controlling the strength of the alloy is the subject of this publication.

## Results

### Tensile property measurement

**Figure 1:**
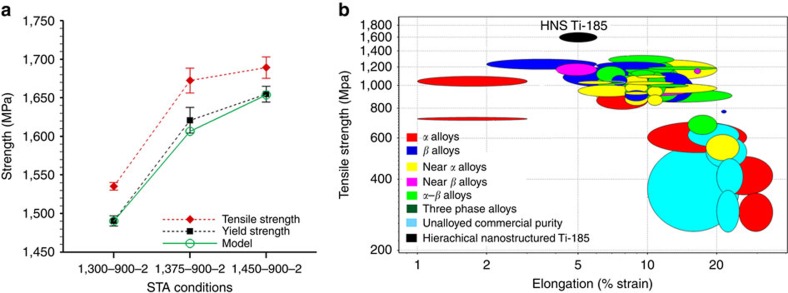
Tensile strength comparison with model and other Ti alloys. (**a**). Ultimate tensile and yield strength of Ti185 for three STA conditions. Also shown in (**a**) is the prediction of empirical model in equation (2). (**b**) Comparison of tensile strength and strain to failure (elongation) of Ti185 alloy with other commercially available Titanium alloys. The graph in (**b**) is plotted using CES Selector software, Granta Design, Cambridge, UK.

The mechanical properties of the specimens characterized in terms of ultimate tensile strength (TS) and 0.2% offset yield strength (YS) subjected to the three solution treated and annealed (STA) conditions are shown in Fig. 1a. As shown in the figure, both the TS and YS of the specimens increased considerably with the increase in solution treatment temperature. In an average the TS increased from ∼1,535 to 1,690 MPa and YS increased from 1,490 to 1,655 MPa for an increase in solution treatment temperature from 1,300 to 1,450 °F. The nominal strain to failure of all the specimens, regardless of the STA condition was approximately in the 4–6% range. The tensile strength of the STA treated low-cost Ti185 alloy produced here is ∼15% greater than the currently available strongest commercial titanium alloy while the strain to failure (elongation) is at par, as shown in Fig. 1b (plotted using CES Selector software, Granta Design, Cambridge, UK). For comparison with a second database of mechanical properties of titanium alloys, as per 2012 ASM Titanium alloy database^16^, the range of ultimate tensile strength of five different class of titanium alloys is as follows, alpha and near alpha alloys (581–1,240 MPa), alpha–beta alloys (827–1,580 MPa), alpha–beta/metastable beta (945 MPa), metastable beta alloys (545–1,573 MPa) and unalloyed and modified Ti (331–662 MPa). From the database, the highest mechanical properties are exhibited by Ti–6Al–2Sn–4Zr–6Mo alloy in STA heat treated condition with 1,580 MPa, the second highest strength was for Ti–15Mo–5Zr–3Al also in STA condition at 1,573 MPa and then the next strongest is Ti–13V–11Cr–3Al alloy with 1,483 MPa (ref. 16). The ultimate tensile strength value of 1,690 MPa achieved by the hierarchically nanostructured Ti185 alloy after 1,450-900-2 STA is higher than the ultimate strength of the strongest titanium alloys in this data base also. It is also noteworthy to mention that these alloys as compared with Ti185 have higher concentrations or additions of expensive elements.

### Microstructure characterization of STA conditions

**Figure 2:**
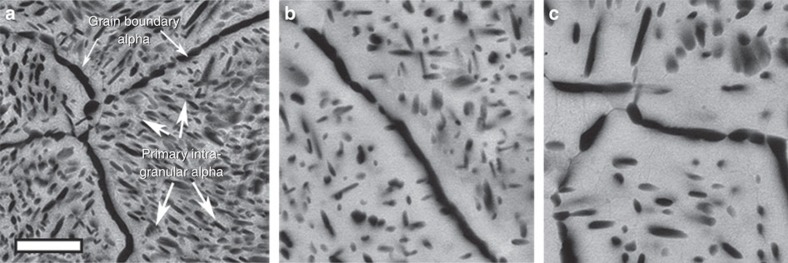
Microstructure of STA conditions. Representative microstructure of the specimens subjected to three STA conditions (**a**) 1,300-900-2, (**b**) 1,375-900-2, and (**c**) 1,450-900-2. Length of scale bar is 5 μm.

To explore the origin of increase in strength with increasing solution treatment temperature we analyzed the microstructure of the specimens subjected to three STA conditions. The representative scanning electron microscopy (SEM) backscattered electron (BSE) images of the three STA conditions 1,300-900-2, 1,375–900–2 and 1,450-900-2 are shown in Fig. 2. In general both grain boundary *α*-phase and intragranular *α*-phase precipitates are present in the specimens corresponding to the three STA conditions. The large intragranular *α*-phase precipitates seen in the SEM BSE images in Fig. 2 will be referred to as primary intragranular *α* in the remainder of this paper. The primary intragranular *α* precipitates in the 1,300-900-2 STA condition have an average length of 2.2 μm and width of 0.4 μm, with an inter-particle spacing of roughly 0.6 μm. In 1,375–900–2 STA condition the average length and width of primary *α* are 2.78 μm and 0.52 μm, respectively, with inter-particle spacing of roughly 0.89 μm. Finally in the 1,450-900-2 STA condition, the primary *α* precipitates on an average are 4.2 μm long, 0.8 μm wide with an inter-particle spacing of roughly 1.4 μm. To this point, for the three STA conditions, it can be noted that (i) the morphology of grain boundary *α* is roughly the same for all three STA conditions; (ii) the aspect ratio of the primary *α* precipitates are also roughly the same and are in the range 5.5–5.25 for all the three STA conditions; and (iii) the number density of primary *α* precipitates decreases significantly with increasing solution treatment temperature.

**Figure 3:**
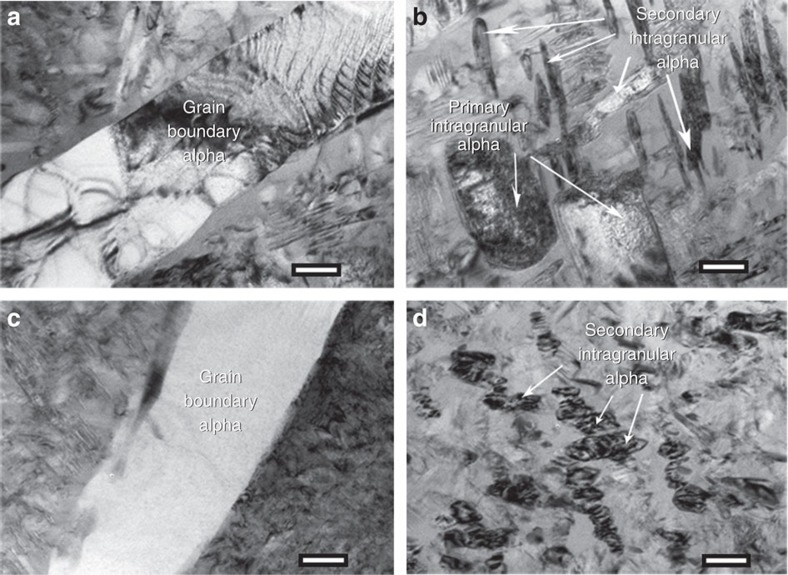
TEM analysis of STA conditions. Bright-field TEM images showing (**a**) grain boundary *α* and intragranular *α* and (**b**) primary and secondary intragranular *α* in a STA 1,300-900-2 specimen. Scale bars are 200 nm in both (**a**–**c**) grain boundary *α* and nanoscale secondary intragranular *α* (scale bar is 200 nm) and (**d**) high-density nanoscale secondary intragranular *α* (scale bar is 100 nm) in a STA 1,450-900-2 specimen.

The decrease in the number density as well as increase in the inter-particle spacing should lead to decrease in the strength^17^, which is in contradiction to the trend observed for tensile strength in Fig. 1b. Hence we carried out transmission electron microscopy (TEM) analysis of the microstructure of the specimens to check the presence of any smaller (nanometer) size scale precipitates. The bright field TEM images of STA condition 1,300-900-2 are shown in Fig. 3a,b, whereas the same for STA condition 1,450-900-2 are shown in Fig. 3c,d. The bright-field TEM images of STA 1,300-900-2 specimen show the presence of grain boundary *α*, primary intragranular *α* and additionally some fine scale *α* precipitates of an average width of 54 nm and length of 386 nm with an inter-particle spacing of roughly 80 nm. This fine scale *α* precipitate will be referred to as secondary *α* precipitate in the remainder of this paper. The secondary *α* precipitates seen in the TEM bright-field images of STA 1,450-900-2 (Fig. 3c,d) are clearly much smaller (34 nm wide and 66 nm long) and with an inter-particle spacing less than 45 nm, and hence much more densely distributed than the secondary *α* in STA 1,300-900-2.

**Figure 4:**
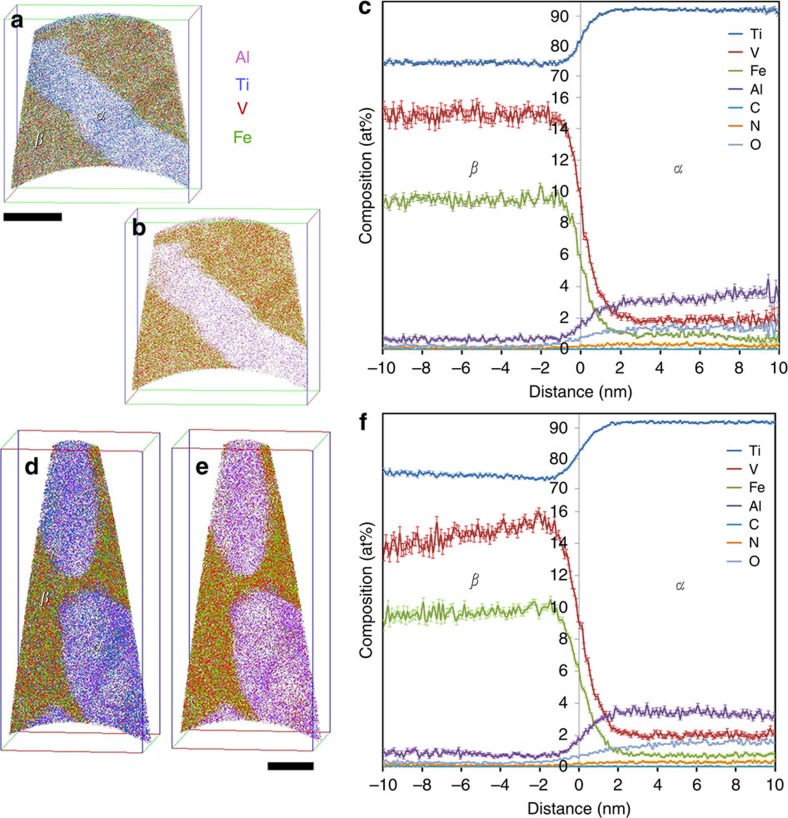
APT analysis of STA conditions. (**a**) All-ionic view of the APT reconstruction showing Ti in blue, V in red, Al in purple and Fe in green, (**b**) ionic view of only Fe, V and Al ions, and (**c**) the solute partitioning between *α* and *β* phase showing V and Fe enrichment in the *β* phase and Ti and Al enrichment in the *α* phase in STA 1,300-900-2 specimen. (**d**) All-ionic view of the APT reconstruction showing the stubby *α* precipitates and the partitioning of Al (purple) from V (red) and Fe (green) is shown in (**e**). The compositional partitioning between *α* and *β* phases in STA 1,450-900-2 (**f**). Scale bars for (**a**,**b**,**d**,**e**) are 20 nm.

Subsequently atom probe tomography (APT) of these alloys was conducted to understand the solute portioning between the *α*- and the *β*-phase. The APT specimens were specifically lifted out from the intragranular regions of the specimens. The APT reconstructions from STA 1,300-900-2 specimen shown in Fig. 4a,b captured a portion of *α*-phase (enriched in Al (purple)) together with *β*-phase (enriched in V (red) and Fe (green)) on either side. The APT reconstruction shows a 63.1 × 63.3 × 63.1 nm^3^ volume within the intragranular region of 1,300-900-2 specimen. From the APT reconstruction, *α* precipitate seems to have a thickness of 20 nm with length and width much larger than the reconstructed volume, pointing to a plate-like morphology. The compositional partitioning between *α*- and *β*-phases is plotted using a proximity histogram^18^ across an 84 at% Ti isocomposition surface (Fig. 4c). The *α* precipitates in the APT reconstructions from STA 1,450-900-2 specimen shown in Fig. 4d,e have smaller size and a discrete morphology in contrast to the plate-like *α* precipitates observed in STA 1,300-900-2 specimen. The APT reconstruction from 1,450-900-2 specimen corresponds to 62.2 × 59.2 × 132.7 nm^3^ volume from the intragranular region. The proximity histogram shown in Fig. 4f shows the corresponding compositional partitioning between *α*- and *β*-phases, and is very similar to elemental partitioning in STA 1,300-900-2 specimen. The steady-state concentration of *α*- and *β*-phases estimated from the steady state regions on either side of both the proximity histograms are given in Table 1. The steady-state compositions of *α*- and *β*-phases in both STA 1,300-900-2 and STA 1,450-900-2 specimens are comparable, which points to attainment of thermodynamically equilibrium solute partitioning during the 2 h aging heat treatment at 900 °F.

### Microstructure characterization of ST conditions

**Figure 5:**
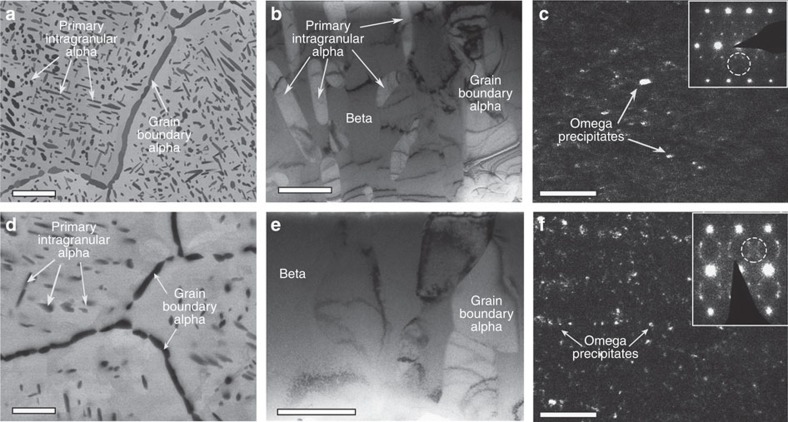
Microstructure of ST conditions. (**a**) SEM BSE image of 1300ST showing grain boundary *α* and primary intragranular *α* (scale bar is 5 μm) (**b**) Bright-field TEM image showing grain boundary *α* and primary intragranular *α* with *β* matrix in between devoid of secondary *α* in a 1,300ST (scale bar is 1 μm); (**c**) dark field image of 1,300ST showing fine scale omega phase distributed within the *β* phase regions (scale bar is 100 nm). Dark field image was formed using omega phase reflections inside the dotted circle in the *β*[113] zone axis SAD shown as inset. (**d**) SEM BSE image of 1,450ST showing grain boundary *α* and primary intragranular *α* (scale bar is 5 μm) (**e**) Bright-field TEM image showing grain boundary *α* and *β* matrix devoid of secondary alpha in 1,450ST (scale bar is 1 μm) (**f**) dark field image showing distribution of fine scale omega phase within the *β* phase regions(scale bar is 100 nm). Dark field image was formed using omega phase reflections inside the dotted circle in the *β*[110]zone axis SAD shown as inset.

To investigate the origin of these hierarchical nanostructures we conducted additional microstructural characterization of the Ti185 alloy subjected only to the solutionizing treatment at 1,300 and 1,450 °F for 1 h followed by water quenching. These conditions will be named as 1,300ST and 1,450ST conditions from here onwards. The SEM BSE image of 1,300ST shows the presence of grain boundary *α* and primary intragranular *α* (Fig. 5a). Bright field TEM image of 1,300ST condition is shown in Fig. 5b where primary *α*-phase precipitates and grain boundary *α* precipitates are marked. Additional distinct reflections arising from omega phase were observed in the [113] zone axis selected area electron diffraction pattern (SAD) of the *β* phase in between primary intragranular *α* phase precipitates (Fig. 5c inset). The *ω* reflections within the dotted circle in SAD were used to form a dark field TEM image highlighting the presence of nanoscale *ω* phase regions in the *β* phase matrix of 1,300ST (Fig. 5c). In comparison the SEM BSE image of 1,450ST clearly shows a much lower number density of intragranular primary *α* precipitates (Fig. 5d). The bright field TEM image of 1450ST only shows presence of grain boundary *α* and *β* phase regions (Fig. 5c). The [110] zone axis SAD of β phase, given in Fig. 5d also shows clear evidence of additional *ω*-phase reflections. The dark field TEM image formed using the *ω*-phase reflections shows the presence of qualitatively larger number density *ω* phase precipitates in the beta phase matrix of 1,450ST condition (Fig. 5d). From these TEM results, it is clear that *ω* phase precipitates are present after solutionizing treatment in 1,300ST and 1,450ST conditions with qualitatively different number densities. Subsequently APT analysis of both 1,300ST and 1,450ST conditions was conducted to quantify the composition of *β* phase regions in between the primary intragranular *α* precipitates. From the APT measured composition of *β* phase given in Table 2, it is clear that the *β* phase in 1,300ST has a higher concentration of *β* stabilizing elements (V and Fe concentration) than in 1,450ST condition.

## Discussion

We now turn to explain the sequence of phase transformation leading to the formation of hierarchical nanostructure in the low-cost powder processed Ti185 specimens subjected to the three STA conditions. The increase in solution treatment temperature increases the stability of the *β*-phase, which explains the decrease in the volume fraction of intragranular primary *α* with increasing solution temperature. This in turn suggests that the large intragranular primary *α* in all three STA treated Ti185 specimens formed during the initial solution treatment stage, which is evident from the significant difference in intragranular primary *α* volume fraction observable in the SEM BSE images between 1,300ST and 1,450ST conditions given in Fig. 5a,d. The Ti185 alloy considered here has the same alloying elements but different concentration as in *β*-titanium alloy, Ti–10V–2Fe–3Al (Ti1023). A number of studies have been reported on varying the morphology and volume fraction of *α*-phase and metastable *ω*-phase precipitates by thermomechanical treatment of Ti1023 alloy^19^,^20^,^21^,^22^. The *ω*-phase was observed in the solution treated and water quenched Ti1023 specimens^23^. The formation of cuboidal shaped metastable *ω*-phase was also reported in another low misfit Ti–V-based alloy upon water quenching past solution treatment^24^. The formation of *ω*-phase in *β*-titanium alloys is in general associated with local rejection of *β* stabilizing elements resulting in local compositional instability^25^,^26^,^27^. This suggests that the microstructure of all three STA treated Ti185 specimens upon water quenching past solution treatment contained: grain boundary *α*, intragranular primary *α*, and retained *β* with *ω*-phase precipitates. The dark field TEM images of Ti185 specimens conditioned to 1,300ST and 1,450ST, Fig. 5c,f, clearly show that such is the case.

The primary *α* formed during the initial solution treatment stage would reject the *β*-stabilizing elements into the remaining *β*-phase hence increasing the solute concentration in the remaining *β*-phase. The high volume fraction of primary *α* precipitates in 1,300ST compared with 1,450ST explains the observed increase in beta stabilizing element concentration in the *β*-phase in 1,300ST (Table 2). The temperature for the start of *ω*-phase transformation during water quenching from *β* or *α*–*β* microstructure was found to be lowered with increasing solute content in the *β*-phase for Ti–V binary alloys^28^, rendering *β* to *ω*-phase transformation unstable for higher solute concentrations in *β*-phase. Hence the *β* to *ω*-phase transformation is comparatively more stable during water quenching past solution treatment at 1,450 °F compared with 1,300 °F due to the lower solute concentration in the *β*-phase. This results in high number density (or say volume fraction) of *ω*-phase precipitates in the retained *β*-phase in the specimens water quenched past solution treatment at 1,450 °F compared with the specimens water quenched past solution treatment at 1,300 °F as qualitatively visible in the dark field TEM images in Fig. 5c,f.

The solution treated water quenched specimens were then subjected to ageing heat treatment at 900 °F for 2 h to complete the STA treatment. The long-term annealing at low temperatures leads to *α*-phase precipitation from existing *ω*-phase precipitates^28^,^29^,^30^,^31^,^32^. For Ti1023 alloy it was suggested in ref. 32 that fine-scale, blocky *α*-phase precipitation occurred from existing *ω*-phase precipitates. Hence the presence of high number density of *ω*-phase precipitates in the specimens solution treated at 1,450 °F resulted in high number density of fine-scale, blocky *α*-phase precipitates past aging treatment at 900 °F. However, the specimens solution treated at 1,300 °F had lower number density of the *ω*-phase precipitates in the retained *β*-phase, hence, comparatively low number density of secondary *α*-phase precipitates nucleated during the aging treatment.

In summary, the final microstructures formed in Ti185 specimens following the three STA conditions have: (i) grain boundary *α*, with roughly similar morphology irrespective of the solution treatment temperature; (ii) primary intragranular *α*, whose number density decreases with increasing solution treatment temperature; (iii) secondary intragranular *α*, whose number density increases with increasing solution treatment temperature; and (iv) *α* and *β*-phases with chemical compositions being independent of the solution treatment temperature. The detailed characterization of both STA and ST conditions permitted to clearly understand the microstructure evolution pathway leading to the final hierarchical nanostructures formed following the three STA conditions. The detailed understanding of the final microstructure for the three STA conditions underlies the understanding of increase in strength with increasing solution treatment temperature.

The roughly similar steady-state partitioning of alloying elements between *α*- and *β*-phase precipitates as well as presence of similar grain boundary *α*-phase precipitates irrespective of the STA condition, suggests that the observed hierarchical nanostructure (two size scales of *α*-phase precipitates) within the grain is the sole factor contributing to strengthening with increasing solution treatment temperature. The effect of bimodal distribution of *α*-phase precipitate on the critical resolved shear stress (CRSS) of the material can be empirically written as^33^,^34^,^35^,^36^:


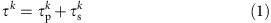


where, *τ* is the overall CRSS of the material, *τ*_p_ is the contribution of primary *α*-phase precipitates,*τ*_s_ is the contribution of secondary *α*-phase precipitates and *k* is an adjustable parameter. The value of *k* lies in the range 1≤*k*≤2 with *k*=1 corresponding to linear superposition and *k*=2 corresponding to the Pythagorean superposition. The STA conditioned Ti185 contains distribution of large primary *α* (more likely to be impenetrable that is, strong) in the midst of fine secondary *α* (more likely to be shearable that is, weak) and for such a microstructure linear superposition is more likely to be dominant^17^,^34^. Next, we know that the critical stress required for a dislocation to overcome obstacles is inversely proportional to the average spacing of the obstacles and the CRSS can be related to the yield strength of the material by the ‘Taylor factor'^17^. Hence, equation (1) under the assumption of linear superposition can be rewritten as:


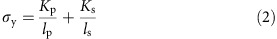


where, *σ*_y_ is the yield strength of the material, *l*_p_ is the inter-particle spacing of the primary *α*-phase precipitates, *l*_s_ is the inter-particle spacing of the secondary *α*-phase precipitates and *K*_p_ and *K*_s_ are constants. Note, all the constants including the ‘Taylor factor' are merged in *K*_p_ and *K*_s_. The values of *l*_p_ for the three STA conditions 1,300-900-2, 1,375-900-2 and 1,450-900-2 in μm are roughly 0.6, 0.89 and 1.4, respectively while the values of *l*_s_ for the three STA conditions in μm are roughly 0.08, 0.054 and 0.045, respectively. The values of the constants *K*_P_ and *K*_s_were found to be 442.6 MPa-μm and 60.2 MPa-μm, respectively. The prediction of the empirical model in equation (2) is compared with the yield strength of the three STA conditioned Ti185 alloy in Fig. 1a and good agreement is noted.

Hence following equation (2) it can be now concluded that the decrease in the number density of primary *α* (increase in inter-particle spacing) with increase in solution treatment temperature decreases the strength of the alloy, which is not only compensated but is surpassed by the increase in the number density (decrease in inter-particle spacing) of fine-scale secondary *α* precipitates. The existence of these two competing changes in the microstructure also explains the relatively small increment in strength by increasing the solution treatment temperature from 1,375 to 1,400 °F compared with the large increment in strength by increasing the solution treatment temperature from 1,300 to 1,375 °F. On the other hand, since intergranular fracture is the dominant failure mechanism in these alloys, the roughly similar strain to failure irrespective of the STA conditions can be dedicated to the uniformity of grain boundary *α*-phase precipitates^37^.

In conclusion, the increase in uniformly distributed, fine-scale secondary *α* precipitates led to the observed increase in strength with increase in solution treatment temperature. Also due to the uniformity of grain boundary *α* precipitates the % elongation of the alloy remained at 4–6% irrespective of the solution treatment temperature. The authors believe that the excellent mechanical properties, when additionally complemented by the low cost of this alloy manufactured by TiH_2_ based powder processing can indeed provide an attractive alternative for replacing more expensive titanium alloys as well as other heavier engineering alloys. We anticipate that this approach can also be used to fabricate other *β*-titanium alloys with hierarchical nanostructure and superior strength.

## Methods

### Ti185 Alloy production and heat treatment

Sintered Ti185 billets produced using low cost TiH2-based powder processes were obtained from Advance Materials Products, Inc., (ADMA) Hudson, Ohio. The billets were then thermo-mechanically processed (hot rod-rolled) to achieve a reduction ratio of 31:1 to form 16 mm diameter rods, for further details see ref. 15. The samples were later solution treated (below the *β* transus temperature, which is 1,526 °F for this alloy) at 1,300, 1,375 and 1,450 °F for 1 h and aged at 900 °F for 2 h. (Note: for convenience degree Fahrenheit is used in the current work instead of degree Celsius). Hereafter this heat treatment condition is abbreviated as STA and the three STA conditions are designated as 1,300-900-2, 1,375-900-2 and 1,400-900-2.

### Tensile testing

The mechanical behavior of 4–5 specimens corresponding to each STA condition were tested under uniaxial tension in a MTS Model 312.21 test frame with a 45kN Lebow 3116 load cell at a constant nominal strain rate of 10^−3^ s^-1^. The extension in the gauge length was measured using a 12.7 mm MTS Model 634.32E-24 extensometer.

### Microstructural characterization

The microstructure of all three STA conditions were characterized using SEM BSE imaging in an FEI Quanta dual beam focused ion beam (FIB) system. The TEM samples and APT samples were also prepared using the FEI Quanta dual beam FIB. TEM imaging was performed using JEOL 2010F transmission electron microscope. The APT specimen preparation method by site specific FIB lift-out and annular milling^38^ aided in selecting specific regions within the intragranular region of the Ti185 alloys. A CAMECA LEAP 4000XHR system equipped with pulsed UV laser (355 nm wavelength) was used to perform APT experiments using 20 pJ laser pulse energy and 0.005 atoms/pulse evaporation rate at specimen temperature of 60 K. The APT results were reconstructed and analyzed using Interactive Visualization and Analysis Software (IVAS) 3.6.8 using standard reconstruction procedure^39^. The reconstruction and composition measurement of all APT data was done using *x*–*y*–z voxels of 1 × 1 × 1 nm size with delocalization of 3 × 3 × 1.5 nm, respectively. For composition measurements, the mass-to-charge spectra peak at 27 Da has an overlap of Al^+1^ and the 54 isotope of Fe^+2^. This peak overlap was de-convoluted using the count of non-overlapping Fe^+2^ isotope counts at 28, 28.5 and 29 Da and estimating corresponding Fe^+2^ counts at 27 Da using the natural abundance of 5.845%. The error bars for composition measurements were estimated based on statistical error for measured atom count as per the equation 
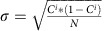
, where *C*^*i*^ corresponds to measured atomic concentration fraction of the element and *N* is the total atom counts in the bin.

## Additional information

**How to cite this article:** Devaraj, A. *et al*. A low-cost hierarchical nanostructured beta-titanium alloy with high strength. *Nat. Commun.* 7:11176 doi: 10.1038/ncomms11176 (2016).

## Figures and Tables

**Table 1:** Phase composition analysis of STA conditions.

**STA ID**	**Phase**	**Measured elemental composition from APT analysis (at %)**						
		**Ti**	**V**	**Fe**	**Al**	**C**	**N**	**O**
1,300-900-2	*α*	91.95±0.27	2.03±0.14	0.75±0.08	3.34±0.18	0.05±0.02	0.33±0.06	1.56±0.12
1,450-900-2	*α*	92.06±0.39	1.91±0.2	0.79±0.12	3.48±0.26	0.05±0.03	0.29±0.08	1.42±0.17
1,300-900-2	*β*	74.93±0.48	14.08±0.39	9.55±0.33	0.9±0.10	0.07±0.03	0.17±0.05	0.3±0.06
1,450-900-2	*β*	74.52±0.46	14.90±0.38	9.49±0.31	0.67±0.09	0.05±0.02	0.14±0.04	0.24±0.05

A comparison of steady-state *α* and *β*-phase composition of STA 1,300-900-2 and STA 1,450-900-2 specimens.

**Table 2:** Beta phase composition analysis of ST conditions.

**ST ID**	**Phase**	**Measured elemental composition from APT analysis (at %)**						
		**Ti**	**V**	**Fe**	**Al**	**C**	**N**	**O**
1,300ST	*β*	79.90±0.05	10.46±0.04	6.52±0.03	1.96±0.02	0.06±0.003	0.33±0.01	0.78±0.01
1,450ST	*β*	82.35±0.04	8.87±0.03	5.74±0.02	2.04±0.01	0.03±0.002	0.15±0.004	0.8±0.01

Beta phase composition of only solutionized and water quenched samples measured from APT analysis.
